# VDAC2 interacts with PFKP to regulate glucose metabolism and phenotypic reprogramming of glioma stem cells

**DOI:** 10.1038/s41419-018-1015-x

**Published:** 2018-09-24

**Authors:** Kai Zhou, Yue-Liang Yao, Zhi-Cheng He, Cong Chen, Xiao-Ning Zhang, Kai-Di Yang, Yu-Qi Liu, Qing Liu, Wen-Juan Fu, Ya-Ping Chen, Qin Niu, Qing-Hua Ma, Rong Zhou, Xiao-Hong Yao, Xia Zhang, You-Hong Cui, Xiu-Wu Bian, Yu Shi, Yi-Fang Ping

**Affiliations:** 1 Institute of Pathology and Southwest Cancer Center, Southwest Hospital, Third Military Medical University (Army Medical University), Chongqing, 400038 China; 2 Key Laboratory of Tumor Immunopathology, Ministry of Education of China, Chongqing, 400038 China

## Abstract

Plastic phenotype convention between glioma stem cells (GSCs) and non-stem tumor cells (NSTCs) significantly fuels glioblastoma heterogeneity that causes therapeutic failure. Recent progressions indicate that glucose metabolic reprogramming could drive cell fates. However, the metabolic pattern of GSCs and NSTCs and its association with tumor cell phenotypes remain largely unknown. Here we found that GSCs were more glycolytic than NSTCs, and voltage-dependent anion channel 2 (VDAC2), a mitochondrial membrane protein, was critical for metabolic switching between GSCs and NSTCs to affect their phenotypes. VDAC2 was highly expressed in NSTCs relative to GSCs and coupled a glycolytic rate-limiting enzyme platelet-type of phosphofructokinase (PFKP) on mitochondrion to inhibit PFKP-mediated glycolysis required for GSC maintenance. Disruption of VDAC2 induced dedifferentiation of NSTCs to acquire GSC features, including the enhanced self-renewal, preferential expression of GSC markers, and increased tumorigenicity. Inversely, enforced expression ofVDAC2 impaired the self-renewal and highly tumorigenic properties of GSCs. PFK inhibitor clotrimazole compromised the effect of VDAC2 disruption on glycolytic reprogramming and GSC phenotypic transition. Clinically, VDAC2 expression inversely correlated with glioma grades (Immunohistochemical staining scores of VDAC2 were 4.7 ± 2.8, 3.2 ± 1.9, and 1.9 ± 1.9 for grade II, grade III, and IV, respectively, *p* < 0.05 for all) and the patients with high expression of VDAC2 had longer overall survival than those with low expression of VDAC2 (*p* = 0.0008). In conclusion, we demonstrate that VDAC2 is a new glycolytic regulator controlling the phenotype transition between glioma stem cells and non-stem cells and may serves as a new prognostic indicator and a potential therapeutic target for glioma patients.

## Introduction

Glioblastoma (GBM) is the most prevalent and malignant primary brain tumor^1,2^. Despite aggressive surgical resection, chemotherapy, and radiotherapy are performed, patients with GBMs still have dismal prognosis, with a median survival < 15 months^3^. Growing evidence supports that GBMs are functionally heterogeneous and harbor a subset of tumor cells with stem cell characteristics, including the preferential expression of stem cell markers, the enhanced self-renewal ability, and multi-lineage differentiation potential^4,5^. Those cells are termed as glioma stem cells (GSCs) and are highly capable of initiating tumor growth or repopulating tumor after treatment^6,7^. Recent studies have demonstrated that GSCs are highly adaptive to various crucial conditions, including nutrient-restricted condition, hypoxia, or chemo-agent exposure^8–10^, and actively interact with the microenvironmental factors to evade antitumor immune responses, promote tumor angiogenesis and tumor invasion^11–13^, thus significantly contribute to tumor recurrence and the poor outcome of GBM patients^14,15^. Despite the pivotal role of GSCs in GBM progression, effective GSC-targeting therapeutics are still unavailable. One major challenge for anti-GSC strategy is that both GSCs and its counterparts non-stem tumor cells (NSTCs) are plastic and capable of undergoing phenotypic transition. Convincing evidence has demonstrated that upon appropriate stimuli GSCs are capable to differentiate into NSTCs to sustain tumor growth^16^. Alternatively, NSTCs could potentiate the acquisition of GSC properties like enhanced self-renewal or highly tumorigenic abilities under certain conditions, thus contribute to therapeutic resistance^17,18^. Therefore, blocking the phenotypic transition between GSCs and NSTCs is of great translational significance to improve the efficacy of GSC-targeting therapy and to benefit GBM patients. However, the intrinsic driving force that determines the plasticity of GSCs and NSTCs is still largely unknown.

Reprogrammed metabolism has been recognized as a hallmark of cancers and fuels multiple biological processes, including cell fate determination^19,20^. Mitochondrial oxidative phosphorylation (OxPhos) and aerobic glycolysis (a phenomenon termed “the Warburg effect”) represent two major metabolic phenotypes for energy production^19^. Although OxPhos-dependent energy production is sufficient to support the maintenance of complex normal cells, most cancer cells are more likely to switch to glycolysis to generate energy and carbon sources needed for tumor growth^20^. GBM is an extremely metabolically active brain tumor that derives energy almost entirely from glucose. However, the metabolic phenotypes of GSCs and NSTCs are still controversial. A predominant view holds that GSCs are less reliant on oxygen-dependent mitochondrial OxPhos than NSTCs, which is supported by the fact that the mitochondria in GSCs are much more fragmented relative to those in NSTCs^21^ and the mitochondrial respiratory activity in GSCs is down–regulated^22^. Instead, GSCs are more dependent on glycolysis to acquire and utilize nutrient in relatively harsh microenvironments^22,23^. Upon nutrient restriction or hypoxia, GSCs exert preferential glucose uptake and redirect the molecular cascade toward glycolysis to become less apoptotic and keep their self-renewal and highly tumorigenic properties^8,24,25^. The lactic acid generated during glycolysis could induce the breakdown of extracellular interstitial matrix thus promote tumor expansion^25^. Intriguingly, heterogeneous GSCs may also switch to additional metabolisms like purine synthesis to self-renew and could be less glycolytic than NSTCs in perivascular tumor microenvironment^24,26,27^. Despite distinct metabolic phenotypes in GBMs, accumulating data indicate that metabolic reprogramming is not simply a passenger, but may be an initiating event in regulating cell plasticity thus fuel the tumor hierarchy^27,28^. It has been reported that nutrient restriction-induced adaptive metabolic changes like preferential glucose uptake could enrich GSCs and may lead NSTCs to acquire of GSC features^8^. Therefore, investigation of key regulators driving the metabolic reprogramming is of great importance to understand GBM heterogeneity and to develop treatments.

Through proteomic screening, we identified that voltage-dependent anion channel 2 (VDAC2), a mitochondrial membrane protein required for cell metabolism, was differentially expressed in GSCs and NSTCs. Previous studies have reported that VDAC family members are β-barrel-forming transmembrane channel proteins^29,30^ and function as governors of mitochondrial bioenergetics to regulate the cross of metabolites like ADP/ATP, NADH flux, and ions through mitochondrial outer membrane (MOM), thus control mitochondrial-dependent metabolism and cell survival^31–33^. Importantly, VDAC2 has been reported as a scaffold protein to combine with multiple partners, including BAK, BECN1/BCL2L1, or hexokinase and localize them on MOM, thus regulate the cellular processes associated with cell apoptosis, autophagy, and glycolysis^34–36^. Moreover, aberrant VDAC2 expression or functioning has been reported in multiple tumors, including melanoma, epithelial thyroid tumors, and breast cancer, indicating that targeting VDAC2 is of therapeutic significance for cancer treatment^37–39^. However, the role of VDAC2 in regulating the metabolic reprogramming and plasticity of GSCs is still unknown.

Herein, we investigated whether VDAC2 is a key regulator to reprogram metabolic phenotypes of GSCs and NSTCs thus affect their plasticity. Disrupting VDAC2 expression in NSTCs reprogrammed glucose metabolism to glycolysis and potentiated the acquisition of GSC features, whereas enforced expression of VDAC2 in GSCs disrupted glycolysis impaired the self-renewal and highly tumorigenic properties of GSCs. As metabolic reprogramming represents a significant driving event for controlling tumor cell plasticity and eventually affecting tumor heterogeneity responsible for therapeutic failure, this study reveals a new glycolytic regulator for the development of new anti-GBM strategies.

## Results

### GSCs are more glycolytic than NSTCs to maintain GSC phenotype

**Fig. 1:**
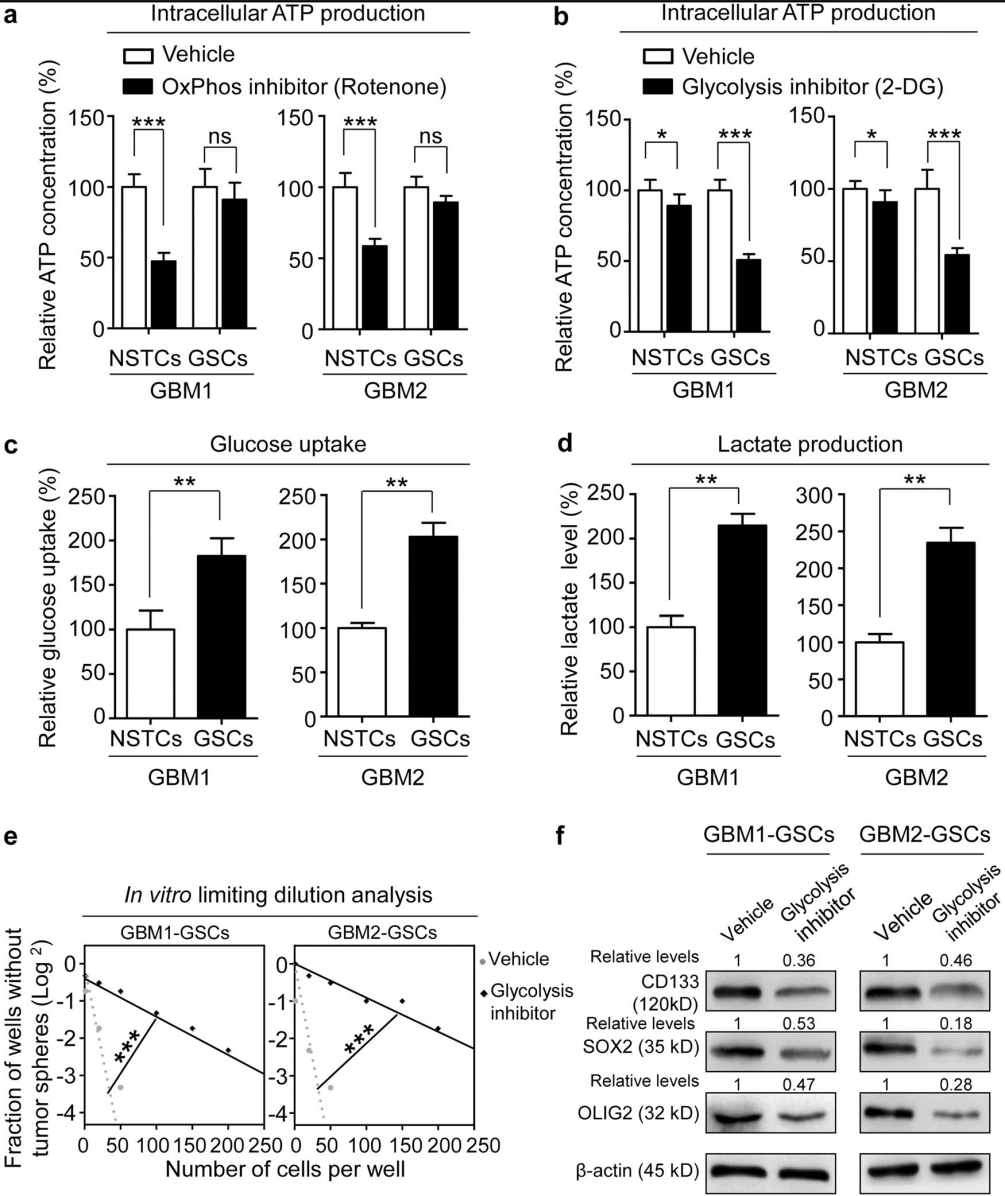
GSCs are more glycolytic than NSTCs to maintain GSC phenotype. **a** Measurement of the intracellular ATP concentrations in GSCs and NSTCs treated with OxPhos inhibitor rotenone or vehicle control. Disrupting OxPhos metabolism by rotenone significantly decreases ATP production in NSTCs but not in GSCs. GSCs and matched NSTCs used in this study were established from two GBM surgical specimens, which were named as GBM1 and GBM2, respectively (***p < 0.001, ns not significant). **b** Measurement of the intracellular ATP concentrations in GSCs and NSTCs treated with glycolysis inhibitor 2-DG or vehicle control. Disrupting glycolytic metabolism by 2-DG robustly reduces ATP production in GSCs but modestly suppresses ATP production in NSTCs (***p < 0.001, *p < 0.05). **c** Analysis of glucose-uptake ratio of NSTCs relative to GSCs. GSCs are more advanced to uptake glucose than NSTCs (**p < 0.01). **d** Analysis of the lactate production in NSTCs relative to GSCs. GSCs produce more lactate, a metabolite of glycolysis, than NSTCs (**p < 0.01). **e** In vitro limiting dilution analysis of GSCs treated with or without glycolysis inhibitor 2-DG. Disrupting glycolytic metabolism by 2-DG inhibits the self-renewal capacity of GSCs (***p < 0.001). **f** Western blot analyses of GSC markers CD133, SOX2, and OLIG2 in GSCs treated with or without glycolysis inhibitor 2-DG. Expressions of CD133, SOX2, and OLIG2 are decreased in GSCs treated with 2-DG

Metabolic state has been demonstrated as an initial event to determine the cell fate of multiple cancer stem cells (CSCs)^40,41^. To investigate the metabolic patterns of GSCs and its counterpart NSTCs, we first determined the reliance of OxPhos and glycolysis as two major glucose metabolic phenotypes for ATP production in two primary GBM-derived GSCs and NSTCs. Disrupting OxPhos metabolism with rotenone, a classical OxPhos inhibitor, significantly decreased ATP level in NSTCs but not in GSCs (Fig. 1a). However, blocking glycolysis with 2-deoxy-d-glucose (2-DG), a widely used glycolysis inhibitor, markedly reduced ATP generation in GSCs but slightly reduced ATP generation in NSTCs (Fig. 1b), suggesting that GSCs are more relied on glycolysis, whereas NSTCs may largely depend on OxPhos, for glucose metabolism. Consistently, two important parameters representing glycolytic activity, glucose uptake, and lactate production were significantly higher in GSCs than in matched NSTCs (Fig. 1c, d). We then investigated whether disruption of glycolytic process could affect GSC maintenance. The in vitro limiting dilution analyses showed that inhibition of glycolysis by 2-DG significantly suppressed the sphere formation ability of GSCs (Fig. 1e). Likewise, the expression of GSC markers CD133, SOX2, and OLIG2 was significantly reduced upon glycolytic disruption (Fig. 1f). These results indicate that GSCs are more relied on glycolysis than NSTCs for the maintenance of CSC features.

### Increased VDAC2 in NSTCs couples PFKP on mitochondrion and inhibits PFKP-mediated glycolysis

**Fig. 2:**
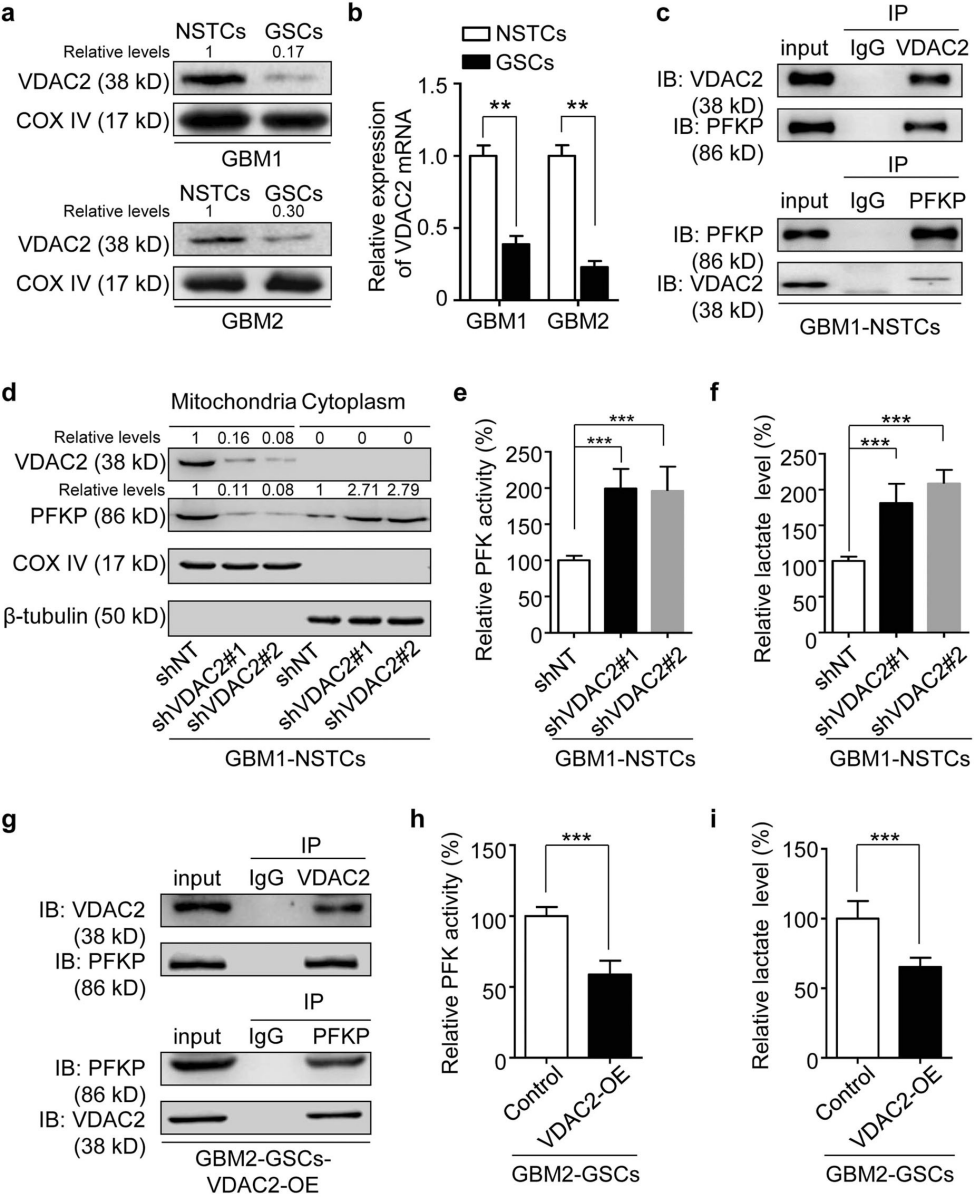
Increased VDAC2 in NSTCs couples PFKP on mitochondrion to prevent its cytoplasmic release and inhibits PFKP-mediated glycolysis. **a** Western blot analyses of VDAC2 expression in GSCs relative to the matched NSTCs derived from human GBMs. COX IV is used as a mitochondrial marker for normalization. **b** qRT-PCR analysis of VDAC2 expression in GSCs and matched NSTCs (**p < 0.01). **c** Co-immunoprecipitation analysis showing the interactions between VDAC2 and PFKP. The anti-VDAC2 antibody (upper panel) and anti-PFKP antibody (lower panel) are used for immunoprecipitation, respectively. The input samples of NSTCs are used as positive controls. **d** Western blot analyses of VDAC2 and PFKP in mitochondrial and cytoplasmic fractions of NSTCs expressing shRNAs against VDAC2 (shVDAC2#1 and #2) or nontargeting shRNA (shNT). COX IV is used as a mitochondrial protein marker and β-tubulin is used as a cytoplasmic protein marker for normalization. Silencing VDAC2 expression reduces the level of PFKP anchored on mitochondrion, but increases PFKP expression in cytoplasm. **e** PFK enzyme activity in NSTCs expressing shVDAC2 or shNT (***p < 0.001). **f** Analysis of the relative lactate production in NSTCs expressing shVDAC2 compared to those expressing shNT (***p < 0.001). **g** Co-immunoprecipitation assay showing the interactions between VDAC2 and PFKP in GSCs expressing VDAC2. The anti-VDAC2 antibody (upper panel) and anti-PFKP antibody (lower panel) are used for immunoprecipitation, respectively. The input samples of GSCs expressing VDAC2 are used as positive controls. **h** Analysis of PFK enzyme activity in GSCs expressing VDAC2 or control vector (***p < 0.001). **i** Analysis of the relative lactate production in GSCs expressing VDAC2 or control vector (***p < 0.001)

To further investigate the mechanism underlying the different metabolic states of GSCs and NSTCs, we performed proteomic screening through mass spectrometric analyses to identify the differentially expressed proteins between GSCs and NSTCs. The top five upregulated and top five downregulated candidates in GSCs relative to NSTCs were listed in Table S2. Of note, VDAC2, a mitochondrial membrane protein required for metabolic regulation, was significantly lower in GBM1-GSCs and GBM2-GSCs than that in matched NSTCs (Fig. 2a). The quantitative real-time PCR (qRT-PCR) results also showed that VDAC2 expression was significantly lower in GSCs than in the matched NSTCs at mRNA level (Fig. 2b). Previous studies have reported that VDAC2 functions as a scaffold protein to combine with multiple kinases and localizes them on mitochondrial membrane, thus regulates their activities^34,35^. Against this backdrop, to investigate whether and how VDAC2 could regulate glycolytic process, we analyzed whether VDAC2 could couple key glycolytic kinases, including PFKP, PKM2, LDHA, and GAPDH to affect their functions. The co-immunoprecipitation analyses showed that VDAC2 interacted with PFKP (Fig. 2c), but not with PKM2, LDHA, or GAPDH in GBM1-NSTCs (Supplementary Figure S1a). Since PFKP functions as a kinase in cytoplasm while VDAC2 is a mitochondrial protein, we proposed that the binding of VDAC2 with PFKP on mitochondrion may limit the release of PFKP into cytoplasm thus affect its activity. To test this possibility, we silenced VDAC2 expression in NSTCs using two different, non-overlapping small hairpin RNA (shRNA) in GBM1-NSTCs (Supplementary Figure S1b) and determined the protein abundance of PFKP in mitochondrial fractions and in cytoplasmic fractions. The western blot results revealed that disruption of VDAC2 reduced the level of PFKP anchored on mitochondrion, but increased PFKP level in cytoplasm compared to control NSTCs expressing nontargeting shRNA (shNT) (Fig. 2d). Consequently, silencing VDAC2 markedly increased the activity of phosphofructokinase (PFK) (Fig. 2e), because the predominant isoform of PFK in human GBMs is PFKP^42^. We next interrogated the impact of VDAC2 on the glycolytic process at the downstream of PFKP, and found that VDAC2 disruption in NSTCs markedly increased the production of lactate, a representative metabolite of glycolysis (Fig. 2f). These results suggest that VDAC2 induces glycolytic reprogramming of NSTCs through affecting PFKP activity.

### Overexpressing VDAC2 in GSCs suppresses PFKP-mediated glycolysis

We next investigated whether VDAC2 could regulate the metabolic reprogramming of GSCs. Because VDAC2 level was lower in GSCs than in NSTCs (Fig. 2a), we overexpressed VDAC2 in GBM2-GSCs (Supplementary Figure S1c) and investigated its effect on PFKP-mediated glycolysis. The co-immunoprecipitation analyses confirmed that overexpressed VDAC2 interacted with PFKP in GSCs (Fig. 2g). Upon VDAC2 overexpressing, the PFK activity and lactate production in GSCs were significantly inhibited (Fig. 2h, i). These results validate that VDAC2 is an important factor to disrupt PFKP-mediated glycolytic process.

### Disrupting VDAC2 in NSTCs potentiates the acquisition of GSC properties

**Fig. 3:**
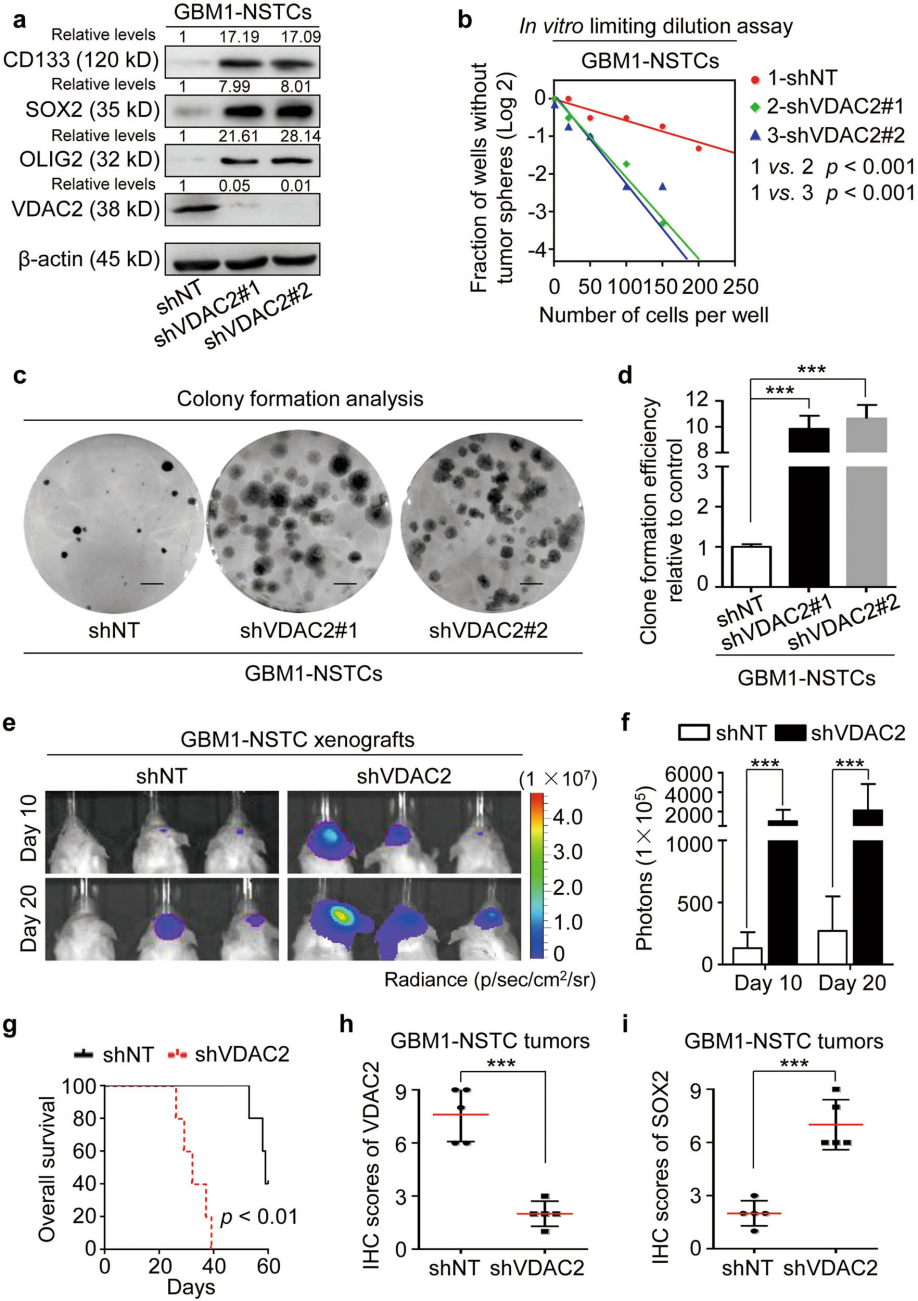
Disrupting VDAC2 in NSTCs potentiates the acquisition of GSC properties. **a** Western blot analyses of the GSC markers (CD133, SOX2, and OLIG2) and VDAC2 in NSTCs expressing shVDAC2 or shNT. The levels of GSC markers CD133, SOX2, and OLIG2 are increased in NSTCs expressing shVDAC2 compared with those expressing shNT. **b** In vitro limiting dilution analysis of the self-renewal capacity of NSTCs expressing shVDAC2 or shNT. Disruption of VDAC2 increases the self-renewal capacity of NSTCs. **c**, **d** Representative images of tumor cell clones (**c**) and quantification of clone formation efficiency (**d**) in NSTCs expressing shVDAC2 relative to those expressing shNT. Silencing VDAC2 expression promotes the clone formation ability of NSTCs (***p < 0.001). **e**, **f** Representative bioluminescent images (**e**) and the quantification (**f**) of xenografts derived from NSTCs expressing shVDAC2 or shNT at day 10 and day 20 after tumor cell implantation. Silencing of VDAC2 markedly promotes tumor formation of xenografts derived from NSTCs. p photons, sr steradian (***p *<* 0.001). **g** Kaplan–Meier survival analysis of mice bearing xenografts derived from NSTCs expressing shVDAC2 or shNT. Silencing of VDAC2 reduces the survival of tumor-bearing mice. *n* = 5/group. **h**, **i** Quantification of the level of VDAC2 (**h**) or GSC marker SOX2 (**i**) in GBM xenografts derived from NSTCs expressing shVDAC2 or shNT by IHC staining (***p *<* 0.001)

Accumulating evidence supported that glycolysis benefits the maintenance of CSC features^8,43,44^. As VDAC2 disruption was important for the glycolytic reprogramming in NSTCs, we investigated whether and how VDAC2 could regulate the phenotypic transition from NSTCs to GSCs. The western blot analyses showed that silencing VDAC2 markedly increased the expression of GSC markers CD133, SOX2, and OLIG2 in GBM1-NSTCs (Fig. 3a). The in vitro limiting dilution analysis and clone formation analysis showed that the ability of sphere and colony formation was significantly enhanced in VDAC2-silenced NSTCs relative to the control NSTCs (Fig. 3b–d), indicating a functional convention from NSTC phenotype to GSC phenotype. Because the enhanced in vivo tumor propagation capacity is the most important feature of GSCs that differs from NSTCs^45,46^, we investigated the effect of VDAC2 disruption on the ability of NSTCs to form xenografts in vivo. The bioluminescence imaging showed that VDAC2-silenced NSTCs exhibited markedly enhanced tumor formation ability compared to control cells (Fig. 3e, f). Kaplan–Meier plots showed that the survival of mice bearing tumors derived from  VDAC2-silenced NSTCs was significantly shorter than that of mice bearing tumors derived from control NSTCs (Fig. 3g). The immunohistochemistry (IHC) analyses confirmed that the expression of VDAC2 was markedly reduced (Fig. 3h and Supplementary Figure S2a), whereas the level of SOX2 was elevated (Fig. 3i and Supplementary Figure S2b) in the xenografts derived from shVDAC2-NSTCs relative to the xenografts derived from control cells. These results indicate that silencing VDAC2 induces NSTCs to acquire GSC features.

### Overexpressing VDAC2 impairs the stem-associated properties of GSCs

**Fig. 4:**
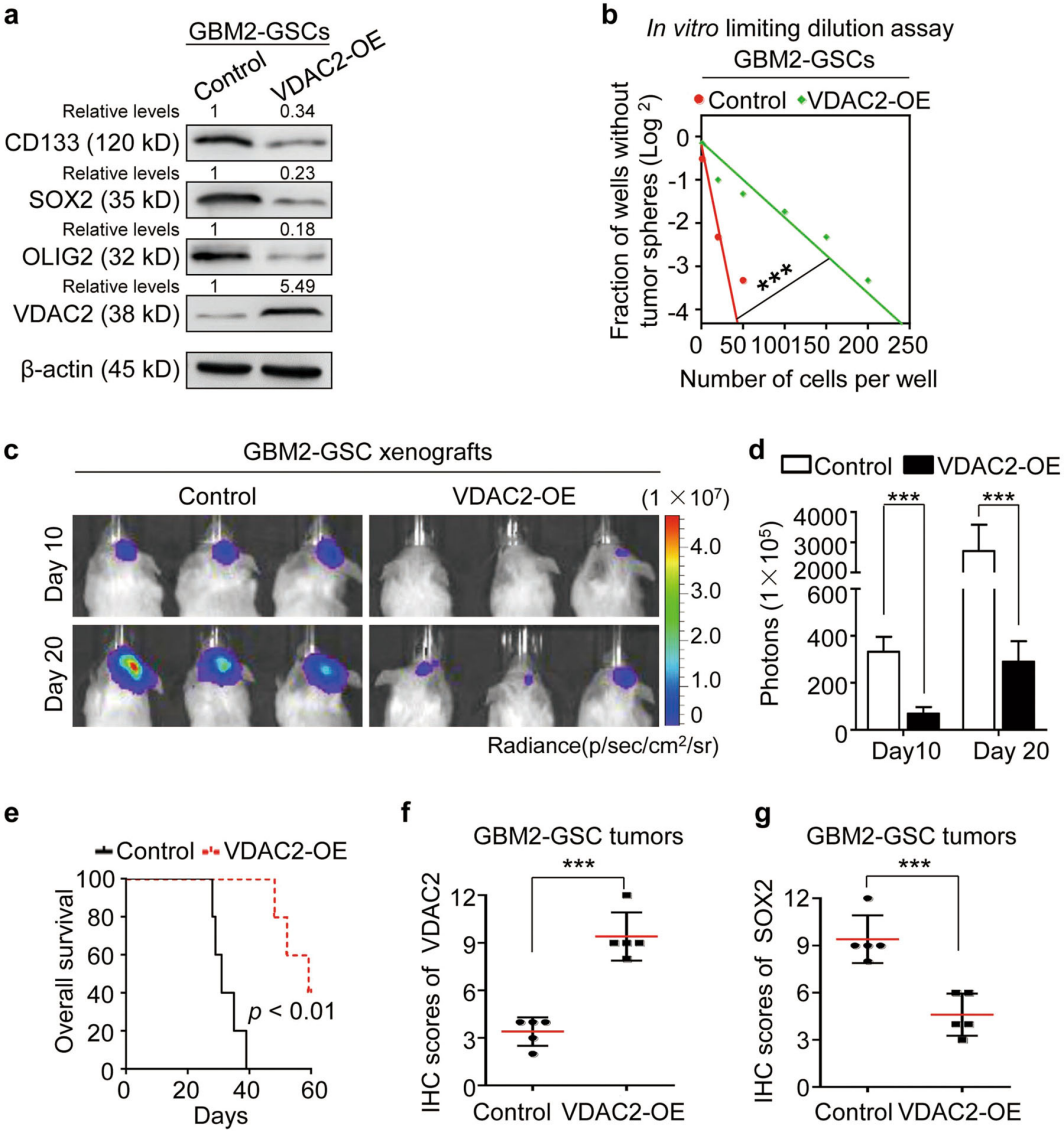
Overexpressing VDAC2 impairs the stem-associated properties of GSCs. **a** Western blot analyses of the GSC markers (CD133, SOX2, and OLIG2) and VDAC2 in GSCs expressing VDAC2 or control vector. **b** In vitro limiting dilution assay of the self-renewal capacity of GSCs expressing VDAC2 or control vector. Ectopic expression of VDAC2 decreases GSC self-renewal capacity (***p < 0.001). **c**, **d** Representative bioluminescent images (**c**) and the quantification (**d**) of xenografts derived from GSCs expressing VDAC2 or control vector at day 10 and day 20 after tumor cell implantation. VDAC2 overexpression markedly suppresses GSC-driven tumor formation. p photons, sr steradian (***p < 0.001). **e** Kaplan–Meier survival analysis of mice bearing xenografts derived from GSCs expressing VDAC2 or control vector. *n* = 5/group. **f**, **g** Quantification of the level of VDAC2 (**f**) or GSC marker SOX2 (**g**) in GBM xenografts derived from GSCs expressing VDAC2 or control vector through IHC staining (***p < 0.001)

To confirm the functions of VDAC2 on regulating GSC plasticity, we overexpressed VDAC2 in GSC populations and determined its effect on the stem-associated properties. The western blot analyses showed that overexpressing VDAC2 disrupted the expressions of GSC markers CD133, SOX2, and OLIG2 in GBM2-GSCs and GBM1-GSCs (Fig. 4a and Supplementary Figure S3a). The in vitro limiting dilution assay confirmed that VDAC2 overexpression significantly inhibited the capability of sphere formation in GBM2-GSCs and GBM1-GSCs (Fig. 4b and Supplementary Figure S3b). Moreover, the tumor formation ability of GBM2-GSCs was potently reduced by enforced VDAC2 expression (Fig. 4c, d). As a consequence, the survival time of mice bearing VDAC2-overexpressing xenografts was significantly extended relative to that of controls (Fig. 4e). The IHC analysis confirmed the upregulated VDAC2 expression (Fig. 4f) and downregulated SOX2 expression (Fig. 4g) in the VDAC2-overexpressing xenografts relative to the control xenografts. These results indicate that VDAC2 overexpression impairs the stem-associated properties of GSCs.

### PFK inhibitor compromises the effect of VDAC2 disruption on glycolytic reprogramming and GSC phenotypic transition

**Fig. 5:**
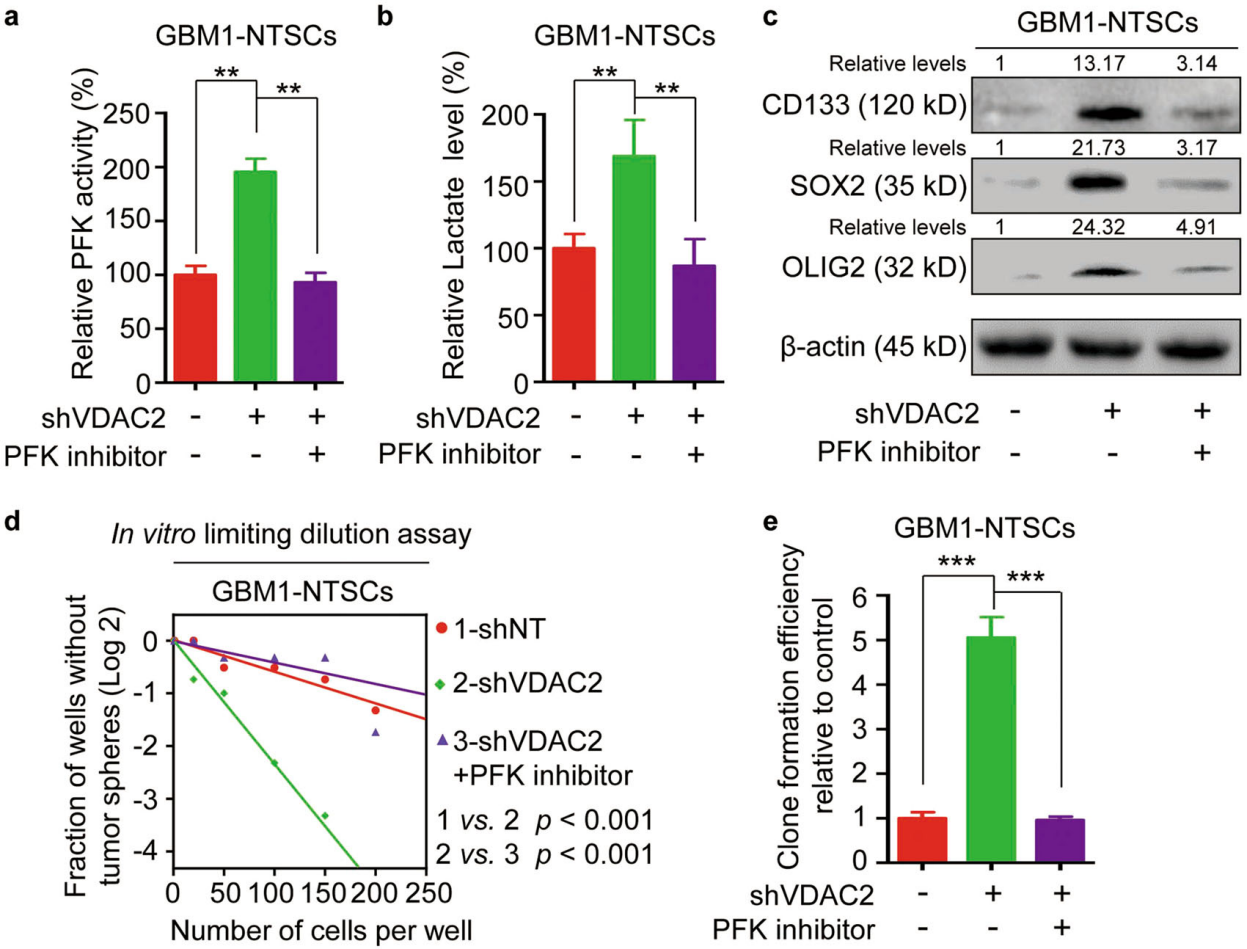
PFK inhibitor compromises the effect of VDAC2 disruption on glycolytic reprogramming and GSC phenotypic transition. **a** Analysis of PFK activity in shVDAC2-expressing NSTCs treated with or without PFK inhibitor CTZ. CTZ treatment impairs the effect of VDAC2 silencing on the promotion of PFK activity (**p < 0.01). **b** Analysis of lactate production in shVDAC2-expressing NSTCs treated with or without PFK inhibitor CTZ (**p < 0.01). **c** Western blot analyses of the GSC markers CD133, SOX2, and OLIG2 in shVDAC2-expressing NSTCs treated with or without PFK inhibitor CTZ. **d** In vitro limiting dilution assay of shVDAC2-expressing NSTCs treated with or without PFK inhibitor CTZ. Inhibition of PFKP by CTZ effectively compromises the VDAC2 disruption-induced self-renewal of NSTCs. **e** Quantification of clone formation efficiency of shVDAC2-expressing NSTCs treated with or without PFK inhibitor CTZ (***p *<* 0.001)

Because PFKP mediated the effect of VDAC2 disruption on glycolysis, we next investigated whether disrupting PFKP function with a PFK inhibitor clotrimazole (CTZ) could compromise VDAC2-knockdown induced glycolysis in NSTCs. CTZ treatment impaired the effect of VDAC2 silencing on the promotion of PFK activity and lactate production in GBM1-NSTCs (Fig. 5a, b). To test whether PFKP inhibition could impair the effect of VDAC2 silencing on GSC phenotypic transition from NSTCs, we compared the expression of GSC markers and the self-renewal ability of shVDAC2-expressing NSTCs treated with or without CTZ. The western blot analysis showed that CTZ treatment significantly attenuated the upregulated expression of GSC markers CD133, SOX2, and OLIG2 induced by VDAC2 silencing (Fig. 5c). The in vitro limiting dilution and clone formation assays indicated that inhibition of PFKP by CTZ effectively compromised the VDAC2 disruption induced sphere and clone formation abilities (Fig. 5d, e). These results demonstrate that PFKP is a major effector of VDAC2 which is responsible for the glycolytic reprogramming and GSC phenotypic transition.

### VDAC2 expression inversely correlates with glioma grades and predicts outcome of glioma patients

**Fig. 6:**
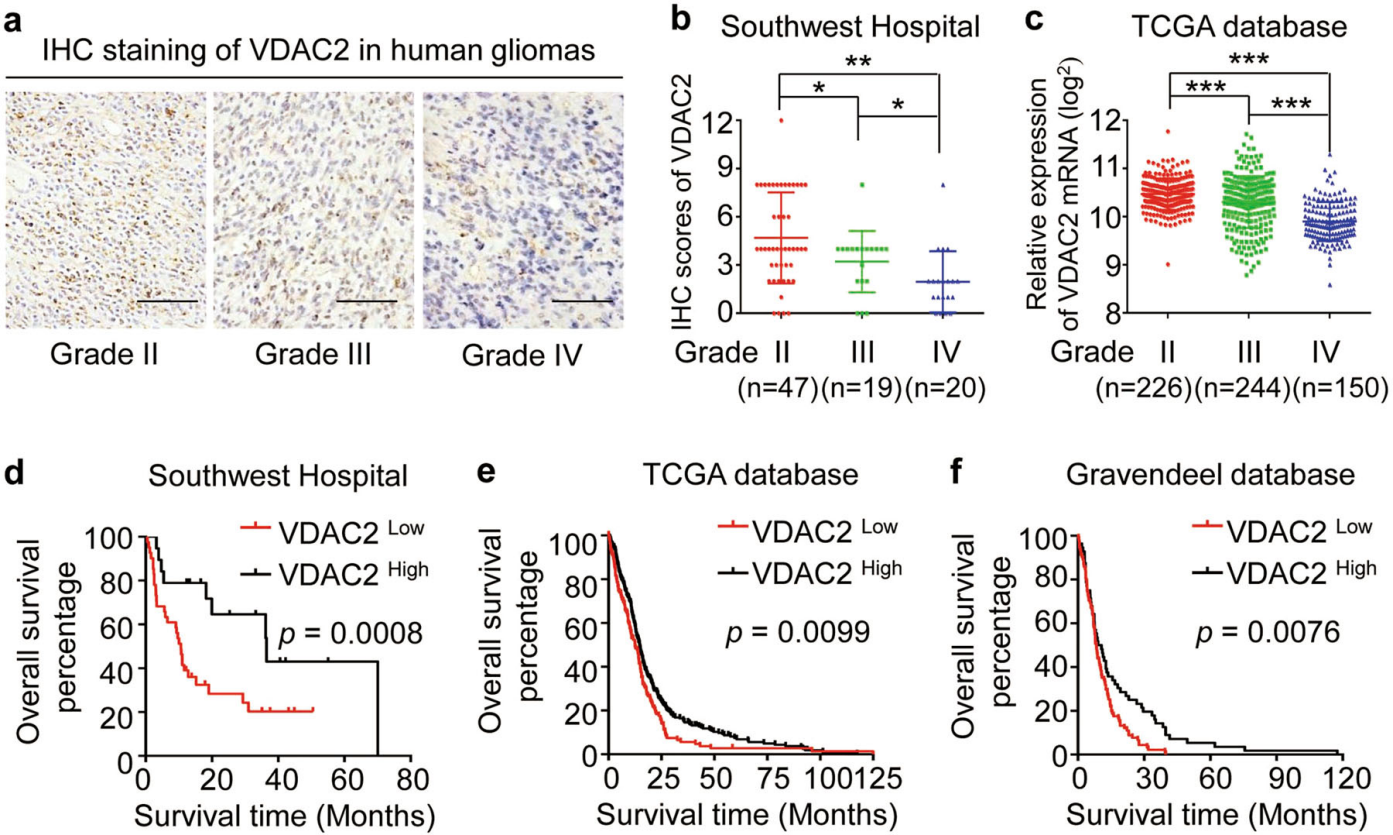
VDAC2 expression inversely correlates with glioma grades and predicts outcome of glioma patients. **a**, **b** Representative IHC images (**a**) and quantification of IHC scores (**b**) of VDAC2 in human gliomas with grade II, grade III, and grade IV (GBM) (**p < 0.01, *p < 0.05). **c** VDAC2 mRNA level in human gliomas with grade II, grade III, and grade IV (GBM) from the TCGA database (***p < 0.001). **d** Kaplan–Meier analysis of VDAC2 expression and overall survival of glioma patients (*n* = 60) from Southwest Hospital. **e**, **f** Kaplan–Meier analysis of VDAC2 expression and overall survival of GBM patients (*n* = 515) from TCGA database (**e**) or those (*n* = 188) from Gravendeel database (**f**). X-tile software is used to determine the cutoff point of VDAC2 expression for the survival analysis

In consideration of important role of VDAC2 in regulating glycolytic reprogramming and GSC phenotypic transition, we investigated the clinical significance of VDAC2 expression in human GBMs. The IHC staining indicated that VDAC2 expression was inversely correlated with glioma grades (Fig. 6a, b), which was confirmed by the data from The Cancer Genome Atlas (TCGA) database (Fig. 6c). Moreover, the Kaplan–Meier analysis showed that glioma patients with higher level of VDAC2 exhibited longer overall survival time, and vice versa (Fig. 6d). The prognostic value of VDAC2 in predicting patient outcomes was further verified in GBM patients from TCGA database and Gravendeel database (Fig. 6e, f). As the proportion of GSC populations significantly correlated with tumor malignancy^47^, examining VDAC2 expression in glioma specimens is of great significant to predict the possibility of GSC phenotypic conversion from NSTCs thus indicates patient outcomes. These results indicate that VDAC2 level inversely correlates with glioma grades and predicts prognosis of GBM patients.

## Discussion

Gliomas are extreme metabolic-active malignant brain tumors^8^. Recent advances support that reprogrammed metabolism could be an initiating event for glioma tumorigenesis and controls the plasticity of tumor cells that responsible for glioma heterogeneity^27,48,49^. Therefore, understanding the mechanisms driving metabolic adaptation and cell fate reprogramming in glioma is important to develop effective therapeutics. Our study demonstrates for the first time that VDAC2 is a critical regulator for the metabolic reprogramming of glioma cells through controlling the PFKP-mediated glycolysis. Moreover, VDAC2 expression is important to mediate the phenotype transformation between GSCs and NSTCs through metabolic switching. Disrupting VDAC2 expression in NSTCs promotes the activation of glycolytic metabolism, thus potentiates the acquisition of GSC properties. Enforced expression of VDAC2 in GSCs suppresses glycolytic activity and disrupts GSC features. Our work highlights metabolic reprogramming as a significant driving event for controlling tumor cell plasticity and eventually affecting tumor heterogeneity of gliomas.

GSCs are a special subset of tumor cells, which are at the top of hierarchy and could generate more differentiated progenies that contribute to the heterogeneity of gliomas. As compared with NSTCs, GSCs are privileged by their unique metabolic process to survive in harsh nutrient-restricted conditions^8^. While there are still discrepancies concerning the metabolic patterns of GSCs, accumulating data strongly supports that glycolysis is often employed by GSCs not only for energy production but also for the generation of glycolytic intermediates necessary to produce biological building blocks (proteins, nucleic acids, and lipids) required for tumor growth^22,24,50^. Besides, the increased concentration of lactic acid and decreased extracellular pH generated during glycolysis could reconstruct GSC microenvironment thus benefits tumor progression^25^. In fact, CSCs derived from many types of tumors, such as osteosarcoma^51^, breast cancer^52^, ovarian cancer^53^, and colon cancer^54^ are much more dependent on glycolysis than the differentiated cancer cells^55^. Our results are in line with previous studies indicating that glycolysis is required for maintaining CSC phenotypes, whereas OxPhos metabolism is required for cell differentiation^25,56^.

We further demonstrate VDAC2-mediated PFKP activity as a new molecular mechanism controlling GSC glycolysis. PFKP is a predominant isoform of PFK that highly expressed in tumors and functions in cytoplasm as a crucial limiting enzyme of glycolytic pathway, which converts fructose-6-phosphate to fructose-1, 6-bisphosphate^57–59^. Previous studies have demonstrated that elevated PFKP activity in GBMs and other solid tumors is associated with increased glycolytic metabolism and is required for the proliferation and tumorigenesis of tumor cells^42,60,61^. Our results enlarged the understanding of PFKP by identifying VDAC2 as a scaffold protein that directly bound to PFKP on mitochondrial membrane to prevent its release into cytoplasm to regulate glycolysis. The loss-of-function data further supported that disrupting VDAC2 expression significantly increased the activity of PFKP thus promoted downstream glycolytic process. Moreover, chemical inhibition of PFKP by CTZ significantly impaired the effect of VDAC2 disruption on glycolytic promotion and the acquirement of GSC features. Therefore, our data reveals VDAC2-mediated PFKP activity as a new metabolic node controlling GSC glycolysis and is helpful for the development of anti-GBM strategies.

Targeting metabolic reprogramming has becoming a promising strategy for cancer treatment. In consideration of the importance of glycolytic adaption for tumor cell surviving, inhibiting specific glycolytic enzymes to suppress or reverse glycolytic reprogramming is crucial to decrease GBM progression. In fact, a number of previous studies have identified GLUT1, PKM2, and LDHA as potential druggable targets for glycolytic reprogramming^62–64^, although there are still challenges in the context of drug specificity, toxicity, or delivery efficacy across the blood–brain barrier. Our study offers VDAC2 and PFKP as potential targets for GBM treatment, allowing for the attack on the interplay between glycolytic metabolism and GSC phenotypic plasticity. Our preclinical study showed that enforced VDAC2 potently disrupted GSC glycolysis, mitigated GSC-driven tumor growth, and extended the survival of tumor-bearing mice. Disruption of PFKP by a molecular inhibitor CTZ significantly blocked the convention from NSTCs to GSCs induced by VDAC2 silencing. This strategy is of potential translational significance, because GSCs are highly resistant to conventional radiation or chemotherapy and the plasticity of GSCs limits the development of specific GSC-targeting treatment. In addition, targeting glycolytic reprogramming may combines with anti-angiogenic treatment  including bevacizumab to generate a synergized effect, because tumor cells could develop a hypoxia-induced glycolytic adaption to overcome anti-angiogenic effect of bevacizumab therapy.

Accumulating evidence supported that the proportion of GSC populations significantly correlated with tumor malignancy and therapeutic resistance^45,47^. Since our functional experiments supported that reduced VDAC2 expression was critical for GSC phenotypic transition from NSTCs, examining VDAC2 expression in glioma specimens is of great diagnostic value to predict the possibility of GSC phenotypic conversion, thus indicates the chance of therapeutic resistance and patient outcome. Indeed, our results showed that high level of VDAC2 expression was associated with better prognosis of patients with gliomas. In summary, our work provides VDAC2 as a new molecular controller for the glycolysis reprogramming and phenotypic conversion between GSCs and NSTCs. Developing therapeutics against glycolysis reprogramming could thus suppress GSC survival and block GSC transition from NSTC to improve management of heterogeneous GBMs.

Notably, although we have demonstrated that VDAC2 is a glycolytic regulator and involves in controlling the phenotype transition between GSCs and NSTCs, the molecular mechanisms of how glycolytic changes regulate the stemness of glioma cells need to be further explored.

## Materials and methods

### Human glioma specimens and cell culture

Glioma specimens were collected from the Neurosurgical Department at Southwest Hospital, Third Military Medical University (TMMU) in accordance with the principles of the Helsinki Declaration, and were approved by the ethics committees of TMMU, with written consents from the patients or their guardians. GSCs and matched NSTCs used in this study were established from two GBM surgical specimens, which were named as GBM1 and GBM2, respectively, as previous described^65,66^. Briefly, tumor cells were isolated from GBM tumors using Papain Dissociation System (Worthington Biochemical). The isolated cells were labeled with a phycoerythrin-conjugated anti-CD133 antibody (Miltenyi Biotec, 130-098-826) and a fluorescein isothiocyanate-conjugated anti-CD15 antibody (Millipore, CBL144F) followed by fluorescence-activated cell sorting to sort GSC population (CD133^+^/CD15^+^) and NSTC population (CD133^−^/CD15^−^). A series of functional assays, including self-renewal analysis (serial neurosphere formation assay), multipotent differentiation analysis (serum-induced multi-lineage differentiation assay), and tumor-initiation analyses (in vivo limiting dilution assay) were applied to validate the CSC phenotypes of the isolated GSCs as previously described^13,65,66^. All the cells were authenticated by karyotype analysis or short tandem repeat profiling and were verified to be free of mycoplasma contamination by PCR analysis. GSCs sorted from GBM xenografts that constantly maintained the GSCs were cultured in Neurobasal medium (12349-015, Gibco) containing epidermal growth factor (10 ng/ml, PeproTech, Rocky Hill, NJ), human recombinant fibroblast growth factor-2 (10 ng/ml, PeproTech), and B27 supplement (20 μl/ml, Life Technologies, Carlsbad, CA), and sorted NSTCs were cultured in Dulbecco’s modified Eagle medium (Gibco) supplemented with 10% fetal bovine serum (Gibco) at 37 °C in 5% CO_2_ and 100% humidity. The cells of three to six passages were used in experiments.

### Isolation of mitochondrial and cytoplasmic fractions

Mitochondrial and cytoplasmic fractions from the indicated tumor cells were isolated using Mitochondria Isolation Kit (MP-007, Invent Biotechnologies, Plymouth, MN) following the manufacturer’s protocol. The isolated mitochondrial and cytoplasmic fractions were used to determine the abundance of indicated proteins in mitochondrion and cytoplasm, respectively.

### Western blot

Western blot analyses were performed as previously described^65,66^. Primary antibodies were listed as follows: anti-VDAC2 (ab47104, Abcam); anti-PFKP (ab204131, Abcam); anti-β-actin (mAb#5125, Cell Signaling Technology, Danvers, MA); anti-COX IV (#4850, Cell Signaling Technology); anti-OLIG2 (ab109186, Abcam, Cambridge, UK); anti-β-tubulin (ab179513, Abcam); anti-CD133 (Miltenyi Biotec, Bergisch Gladbach, Germany); and anti-SOX2 (#3579, Cell Signaling Technology). Secondary antibodies are horseradish peroxidase (HRP)-labeled goat anti-rabbit IgG (H + L) (A0208, Beyotime, Shanghai, China) and HRP-labeled goat anti-mouse IgG (H + L) (A0216, Beyotime). Immuno-bands were quantified by densitometry using ImageJ software (NIH, Bethesda, MD, USA).

### Co-immunoprecipitation

Co-immunoprecipitation was carried out using the Thermo Scientific Pierce Co-IP Kit (26149) following the manufacturer’s protocol. Briefly, anti-VDAC2 (ab126120, Abcam), anti-PFKP (ab204131, Abcam), or an IgG control antibody (mAb#3900, Cell Signaling Technology) was immobilized for 2 h using AminoLink Plus coupling resin. The resin was then washed and incubated with cell lysate overnight. After incubation, the resin was washed again and proteins were eluted using elution buffer. Samples were analyzed by western blot using antibodies, including anti-VDAC2 (ab126120, Abcam), anti-PFKP (ab204131, Abcam), anti-PKM2 (ab131021, Abcam), anti-LDHA (#3582, Cell Signaling Technology), and anti-GAPDH (ab181602, Abcam).

### Quantitative real-time PCR

qRT-PCR was performed as previously described^66^. The sequences of the primer sets for *VDAC2* and *ACTB* (gene encoding β-actin) were listed in Table S1. The mRNA level of* ACTB* was used for normalization.

### In vitro limiting dilution assay

GSCs or NSTCs were seeded into a 96-well plate at a density of 1, 20, 50, 100, 150, or 200 cells/well and culture for 7 days. Twenty replicates were conducted for each concentration. The numbers of cell spheres in each well were calculated and the sphere formation efficiency was calculated using extreme limiting dilution analysis (http://bioinf.wehi.edu.au/software/elda/) as previously described^66^.

### Clone formation assay

Cells were seeded into six-well plates (200 cells/well, 5 wells for each group) and cultured for 15 days. Clones were then stained with crystal violet for 20 min and were imaged using ChemiDoc MP Imaging System (Bio-Rad). Clones with diameter > 75 μm were considered as positive clones and counted. The clone formation efficiency (%) = number of clones/number of seeded cells × 100%.

### PFK activity analysis

PFK activity was assessed using Phosphofructokinase Activity Colorimetric Assay Kit (MAK093, Sigma) according to the manufacturer’s instruction. In all, 2 × 10^6^ of indicated cells were used for this experiment and the PFK activity is calculated by the absorbance of 450 nm using Multiscan GO spectrophotometer (Thermo Fisher Scientific, Vantaa, Finland).

### Glycolytic metabolite analysis

Cells were cultured in fresh medium for 24 h and then the medium was collected and analyzed for the level of glucose or lactate remaining in the medium using a QuantiChrom Glucose Assay Kit (Bioassay Systems, Hayward, CA) or EnzyChrom L-Lactate Assay Kit (Bioassay Systems) following the manufacturer’s guideline. CTZ (C2867, TCI, Tokyo, Japan) was used as a specific PFK inhibitor for the rescue experiments.

### ATP analysis

Intracellular ATP was detected using an ATP Assay Kit (Beyotime Biotechnology, Jiangsu, China) following the manufacturer’s protocol. Briefly, cells were treated with 2-DG (25 mM, D0051, TCI) or rotenone (50 μM, ab143145, Abcam, Cambridge, UK) for 1 h. The indicated cells were lysed and centrifuged at 12 000 × *g* for 5 min at 4 °C. The supernatants were then mixed immediately with dilution buffer containing luciferase. The luminance was determined by using a Luminometer (Promega, Sunnyvale, CA). The concentration of ATP was calculated according to a standard curve and normalized by the cellular protein levels among groups.

### Lentiviral vector construction

The human VDAC2-specific shRNA vectors, the shNT vector, the VDAC2-expressing vector, and control vector were purchased from HANBIO, China. Cells stably expressing shVDAC2 were enriched by puromycin treatment for selecting positive clones.

### Two-dimensional electrophoresis and mass spectrometric analyses

Two-dimensional electrophoresis (2-DE) was conducted with IpGphor II apparatus (Amersham Biosciences, Arlington Heights, IL) as previously mentioned^67^. Protein lysates (150 μg/sample) from NSTCs and GSCs were subjected to 2-DE, followed by silver staining and gel scanning using a GS-800 calibrated densitometer (Bio-Rad, Richmond, CA). Images of gels were analyzed using Quantity One and PDQuest softwares (version 8.0, Bio-Rad). Protein spots with significant different expressions between GSCs and NSTCs (≥10.0-folds) were manually excised from the 2-DE gels and were identified using matrix-assisted laser desorption/ionization time-of-flight mass spectrometry analysis (BGI-Shenzhen, China). Peptide mass mapping was performed by using the program MASCOT (Matrix Science, London, UK) in Swiss-Prot database with a GPS explorer software (Applied Biosystems).

### Immunohistochemistry

Immunohistochemical staining was performed using Dako REALTM EnVision^TM^ Detection System (K5007, Dako, Glostrup, Denmark) in accordance with the manufacturer’s protocol. Rabbit anti-human VDAC2 polyclonal antibody (ab126120, Abcam) and mouse anti-human SOX2 (ab171380, Abcam) were used as the primary antibodies. Immunohistochemical scores of VDAC2 and SOX2 were calculated independently by two histopathologists in a blinded manner according to the staining intensity and percentage of positive tumor cells^68^. The best predictive cutoff value of VDAC2 expression was determined to be 6 analyzed by X-tile software. The score ≥ 6 was defined as VDAC2^high^, otherwise was defined as VDAC2^low^.

### Orthotopic xenografts and bioluminescence imaging

GSCs expressing VDAC2 or NSTCs expressing shVDAC2 or control vector were transfected with luciferase-reporter vector. The abovementioned tumor cells (1 × 10^5^ cells/mouse) were orthotopically implanted into the brains of 6-week-old male SCID mice (Laboratory Animal Center, Southwest Hospital, China). Tumor growth was detected and quantified at day 10 and day 20 after injected by bioluminescence imaging using the In Vivo Imaging System (IVIS, PerkinElmer) and Living Image Software (PerkinElmer). Mice were sacrificed at the indicated time points or until manifestation of neurological symptoms. The animal experiments were approved by the Institutional Animal Care and Use Committee of the Southwest Hospital, TMMU, in accordance with the Guide for the Care and Use of Laboratory Animals.

### Statistical analysis

Statistical analyses were conducted using SPSS 18.0 (SPSS Inc., Chicago, IL) and GraphPad Prism v.6 (La Jolla, CA). The unpaired two-group comparison and multiple comparisons were made using the Student *t*-test or one-way analysis of variance, respectively. Survival analysis was carried out using the Kaplan–Meier method, with the log-rank test for comparison. X-tile software (Yale University, New Haven, CT) was used to determine the cutoff point of VDAC2 expression for the survival analysis^69^. Data were presented as the mean ± SD. *p < 0.05, **p < 0.01, or ***p < 0.001 was considered statistically significant. All experiments were performed independently at least three times.

## Electronic supplementary material

Supplementary information
