# Supplementary material for: VDAC2 interacts with PFKP to regulate glucose metabolism and phenotypic reprogramming of glioma stem cells
Source: Cell Death Dis. 2018 Sep 24;9(10):988. doi: 10.1038/s41419-018-1015-x (PMC6155247; doi:10.1038/s41419-018-1015-x)
Supplement: Supplementary file 1 — Supplementary information [file 41419_2018_1015_MOESM1_ESM.pdf]

Supplementary information

Supplementary Figure 1.

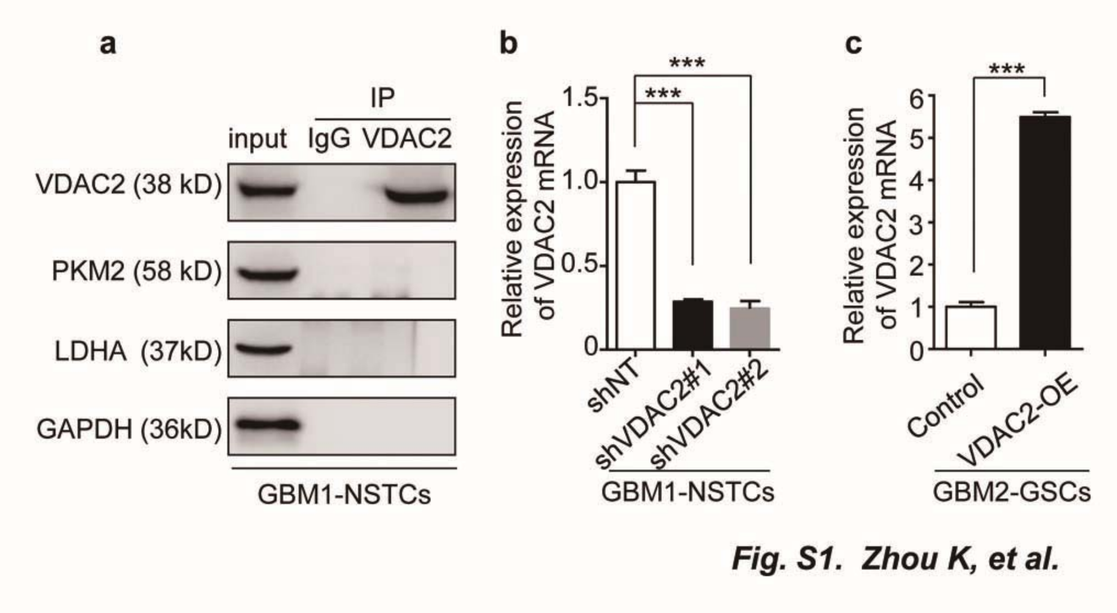

Supplementary Figure 1. VDAC2 silencing or overexpressing efficacy determination and co-immunoprecipitation analyses of the interactions between VDAC2 and PKM2, LDHA, as well as GAPDH.

a Co-immunoprecipitation analyses of the interactions between VDAC2 and PKM2, LDHA, or GAPDH in non-stem tumor cells (NSTCs). The anti-VDAC2 antibody was used for immunoprecipitation. The input samples were used as positive controls. VDAC2 does not interact with PKM2, LDHA, or GAPDH in NSTCs. **b** qRT-PCR analysis of VDAC2 expression in NSTCs expressing shRNAs against VDAC2 (shVDAC2-1, -2) or nontargeting shRNA (shNT) (***$p < 0.001$). **c** qRT-PCR analysis of VDAC2 express in GSCs expressing VDAC2 or control vector (***$p < 0.001$).

Supplementary Figure 2.

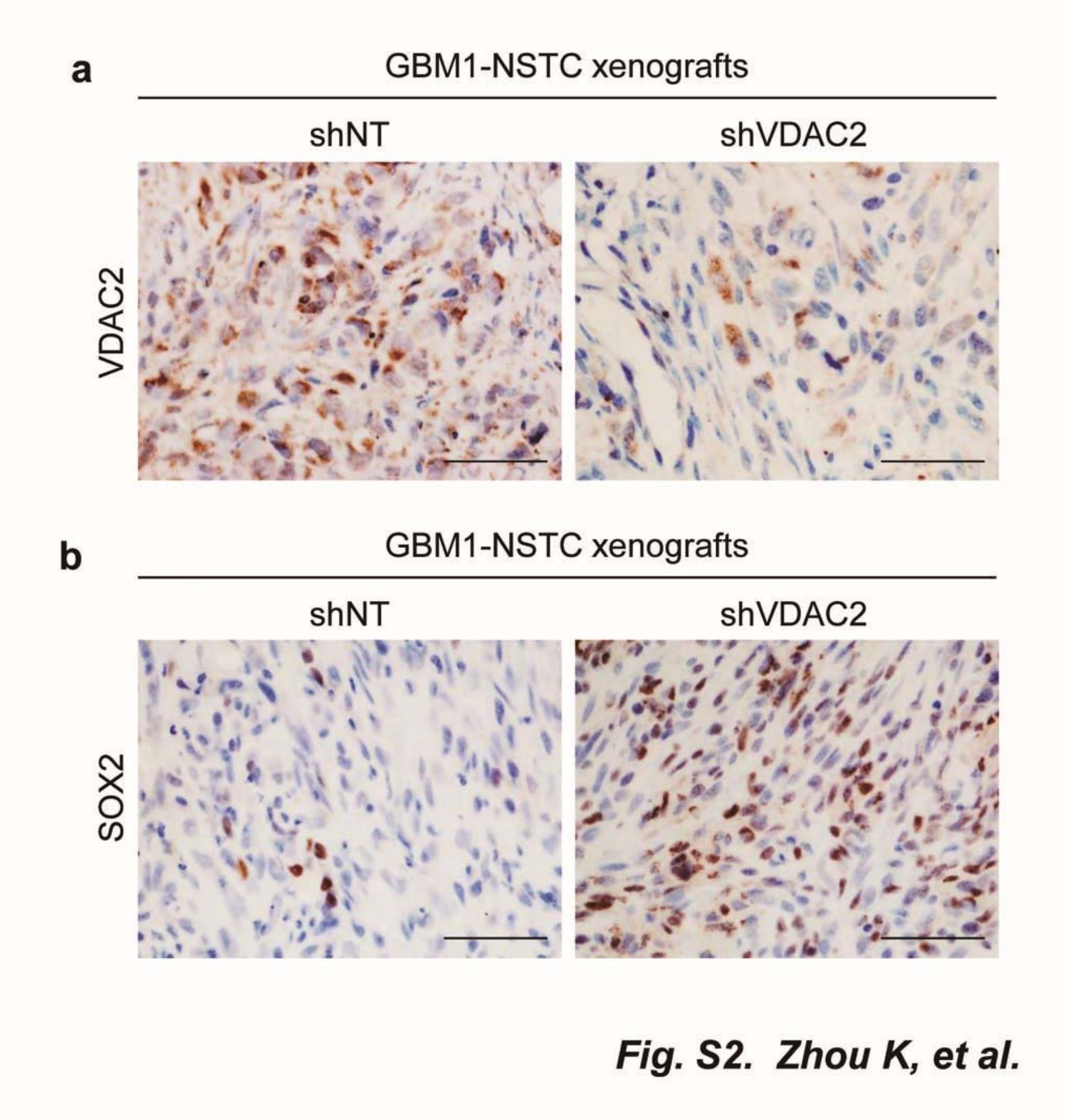

Supplementary Figure 2. Disrupting VDAC2 expression increases the proportion of GSCs marked by SOX2 in GBM xenografts.

a Representative IHC images of VDAC2 in the GBM xenografts derived from NSTCs expressing shVDAC2 or shNT. Scale bar = 100 μm. **b** Representative IHC images of SOX2 in the GBM xenografts derived from NSTCs expressing shVDAC2 or shNT. Scale bar = 100 μm.

Supplementary Figure 3.

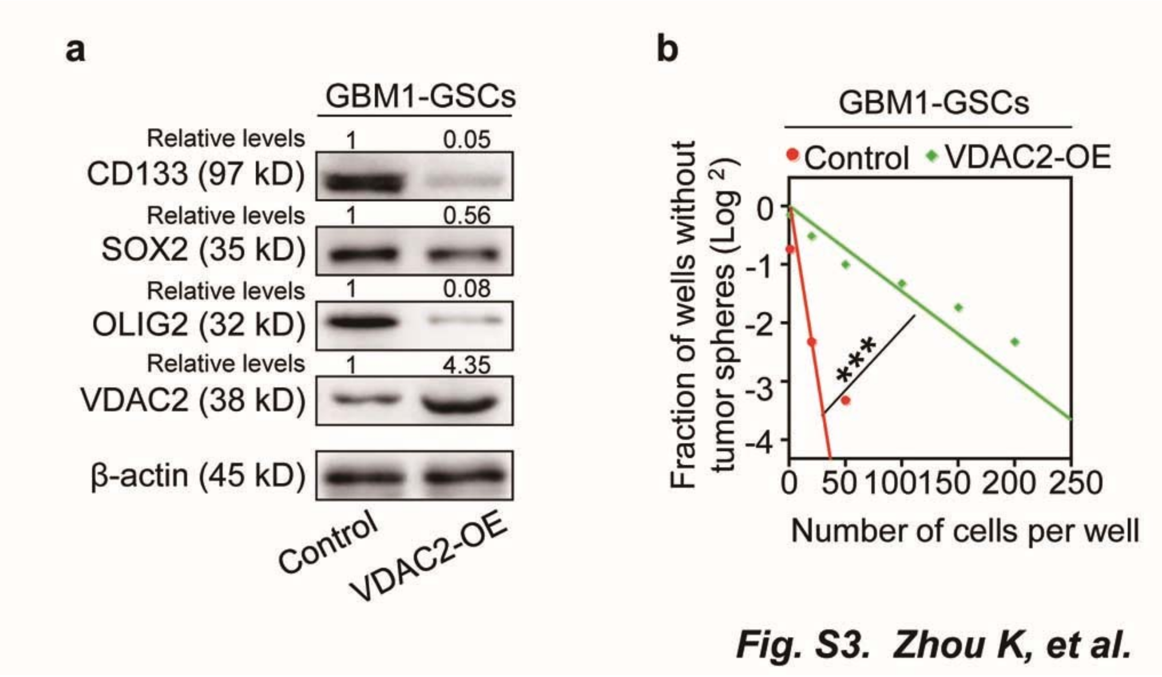

Supplementary Figure 3. Enforced VDAC2 suppresses the expressions of stem cell markers and the self-renewal of GSCs.

a Immunoblot analyses of the expressions of stem cell markers CD133, SOX2 and OLIG2 in GSCs expressing VDAC2 or control vector. **b** *In vitro* limiting dilution analyses of GSCs expressing VDAC2 or control vector. Ectopic expression of VDAC2 decreases GSC self-renewal capacity (***$p < 0.001$).

Table S1. Sequences of primers for qRT-PCR analyses.

Genes	Primer sequences
VDAC2	F: GCTACAGGACTGGGGACTTC
	R: AATGCCAAAACGAGTGCAGTT
ACTB	F: TCATGAAGTGTGACGTTGACA
	R: CCTAGAAGCATTTGCGGTGCAC

Table S2. The top five up-regulated and top five down-regulated proteins in primary non-stem tumor cells (NSTCs) relative to the matched glioma stem cells (GSCs) by two-dimensional gel electrophoresis combined with mass spectrometry.

Protein ID	Protein name	Protein mass (Da)	Coverage rate	Ratio (NSTC/GSC)	Isoelectric point
SSP8302	VDAC2	31972.71	54.95%	Up	7.65
SSP6306	ANXA2	38807.9	45.72%	Up	7.91
SSP1402	TPM1	32827.79	42.25%	Up	4.4
SSP5504	ACTG1	42107.92	28.53%	Up	5.16
SSP5502	CNN3	36561.85	27.66%	Up	5.92
SSP6211	PARK7	20049.55	48.68%	Down	6.78
SSP4818	DNM2	98190.25	26.90%	Down	7.52
SSP6210	UQCRFS1	29934.47	24.45%	Down	8.46
SSP5407	PPP1R12B	110768.3	21.79%	Down	5.37
SSP5801	ALB	46441.89	9.60%	Down	6
